# A 3D-Printed Ceramics Innovative Firing Technique: A Numerical and Experimental Study

**DOI:** 10.3390/ma16186236

**Published:** 2023-09-15

**Authors:** Tiago Santos, Melinda Ramani, Susana Devesa, Catarina Batista, Margarida Franco, Isabel Duarte, Luís Costa, Nelson Ferreira, Nuno Alves, Paula Pascoal-Faria

**Affiliations:** 1 CDRSP—Centre for Rapid and Sustainable Product Development, Polytechnic of Leiria, 2430-028 Marinha Grande, Portugal; melinda.ramani@gmail.com (M.R.); catarina.batista@ipleiria.pt (C.B.); margarida.franco@ipleiria.pt (M.F.); martins.ferreira@ipleiria.pt (N.F.); 2 ARISE—Associated Laboratory on Advanced Production and Intelligent Systems, 4050-313 Porto, Portugal; 3 I3N and Department of Physics, University of Aveiro, 3810-193 Aveiro, Portugal; susanamdevesa@ua.pt (S.D.); kady@ua.pt (L.C.); 4 CEMMPRE—Centre for Mechanical Engineering, Materials and Processes, Department of Mechanical Engineering, University of Coimbra, Rua Luís Reis Santos, 3030-788 Coimbra, Portugal; 5 TEMA—Centre for Mechanical Technology and Automation, Department of Mechanical Engineering, University of Aveiro, 3810-193 Aveiro, Portugal; isabel.duarte@ua.pt; 6 LASI—Intelligent Systems Associate Laboratory, 4800-058 Guimarães, Portugal; 7 Mathematics Department, School of Management and Technology, Polytechnic of Leiria, 2411-901 Leiria, Portugal; 8 Mechanical Engineering Department, School of Management and Technology, Polytechnic of Leiria, 2411-901 Leiria, Portugal

**Keywords:** 3D printing, ceramics, stoneware, numerical analysis, sintering technology, microwave firing

## Abstract

Additive manufacturing (AM), also known as three-dimensional (3D) printing, allows the fabrication of complex parts, which are impossible or very expensive to produce using traditional processes. That is the case for dinnerware and artworks (stoneware, porcelain and clay-based products). After the piece is formed, the greenware is fired at high temperatures so that these pieces gain its mechanical strength and aesthetics. The conventional (gas or resistive heating elements) firing usually requires long heating cycles, presently requiring around 10 h to reach temperatures as high as 1200 °C. Searching for faster processes, 3D-printed stoneware were fired using microwave (MW) radiation. The pieces were fired within 10% of the conventional processing time. The temperature were controlled using a pyrometer and monitored using Process Temperature Control Rings (PTCRs). An error of 1.25% was calculated between the PTCR (1207 ± 15 °C) and the pyrometer (1200 °C). Microwave-fast-fired pieces show similar mechanical strength to the references and to the electrically fast-fired pieces (41, 46 and 34 (N/mm^2^), respectively), presenting aesthetic features closer to the reference. Total porosities of ~4%, ~5% and ~9% were determined for microwave, electrically fast-fired and reference samples. Numerical studies have shown to be essential to better understand and improve the firing process using microwave radiation. In summary, microwave heating can be employed as an alternative to stoneware conventional firing methods, not compromising the quality and features of the processed pieces, and with gains in the heating time.

## 1. Introduction

### 1.1. Summary of 3D Printing

In the context of ceramic manufacturing, piece shaping or forming is usually carried out using methods such as injection molding, casting, high and isostatic pressure and roller process, allowing for the production of high-performance ceramic parts and dinnerware products on a mass production basis [1,2]. Novel fabrication methods, such as 3D printing brings new prospects, offering new and differentiated products, allowing the manufacturing of complex parts, including hollow and framed/latticed structured objects impossible to produce using conventional fabrication techniques [2,3,4,5,6]. Application of 3D printing methods strive to achieve weight reduction, as well as optimization and usage of raw materials [7]. Moreover, it has the advantage of fast prototyping, as opposed to the current form of ceramic prototyping, which is a hand-made and time-consuming process [8]. The manufacturing of decorative ceramic vases and figurines, usually manufactured in reduced numbers and employing gypsum molds and casting processes, also benefits from direct piece forming using AM [9].

With the latest advances in materials and computer sciences, a great variety of 3D printing processes have been developed for ceramic manufacturing [2], such as layer-wise slurry deposition 3D printing (LSD-printing), binder jetting and direct writing (DW), also known as robocasting [3,7,10]. They are extrusion-based methods that use a viscous material, referred to as a “paste” or an “ink”, that are extruded through the nozzle forming individual layered lines [10,11]. With this technology, consumers can interact and customize their own products [12].

Briefly presenting some of the literature, Zocca et al. [4] described the application of LSD-printing to alumina as an example of the potential of AM. Parts of complex-shaped alumina were printed with remarkable definition. These showed high green and sintered densities, with a good comparison to reference parts produced from normalized forming technologies (i.e., uniaxial pressing). Lima et al. [8] studied the 3D printing of porcelain with complex shapes by LSD-printing. The LSD-printing was demonstrated as a novel AM process for the shaping of porcelain samples with complex geometry, good detail resolution and surface finish. This method allowed the production of samples with density, microstructure and mechanical properties comparable to slip-casted porcelain. The authors concluded that the LSD-printing method shows potential to be employed in the silicate ceramics industry, together with conventional forming technologies used for the production of prototypes and series of small products. Paoletti [13] presented the case study of 3D printing technology implemented in the design and mass customization for the clay brick industry. Choi et al. [9] studied the manufacture of porcelain figurines by powder bed-binder jetting 3D printing. The main raw constitutions are 34% dry glass beads, composed of soda-lime silicate (softening point of 730 °C), 46% clay and 20% quartz. After mixing the raw material powders, the 3D-printed parts are bi-fired; first, biscuit firing at 1100 °C, followed by glaze application and a second firing at 1200 °C. The figurines have less than 17 cm and were successfully produced, presenting properties comparable to conventionally produced parts, even considering their lower density and higher water absorption. One of the reasons for these properties was attributed to the lower shear force during the 3D printing of the figurines. 

Balani et al. [14] presented a review of DW printing processes, materials, applications and the current state of the art in several industries. According to the authors, DW is an emerging technology that allows the manufacture of complex, functional and high-end precision macron-scale parts. Among all the DW processes, extrusion-based, energy-assisted and droplet-based technologies, robocasting is the most common and cost-efficient, enabling the manufacturing of a wide range of dimensions scaling from sub-micron to several millimeters for different applications, such as biomedical, electronics and in situ prosthesis.

The relationships between the volume fraction of solids of the clay paste inks, their rheological properties and the appropriate printing parameters were explored by Chan et al. [5]. The authors claimed that a better understanding of the relationship between the process, properties and performance would be an incentive to the traditional ceramics industry towards the adoption of DW technology. Their investigation revealed that the required pressure–speed ratio increased with the increasing of the rheological properties, specifically storage modulus and yield stress. The pressure–speed ratio showed an increasing trend with the decrease in the nozzle size. Hu et al. [15], along with a literature overview on 3D printing methods, presented a mathematical model of the extrusion behavior of the ceramic paste. The extrusion flow rate, related to the nozzle diameter, layer height, paste extrusion speed, scanning speed, paste viscosity, shear stress and compaction and liquid phase migration effects (paste rheologic properties in general), are referred to as several of the parameters that influence the piece extrudability, shape retention, deformation, voids and crack formation. These parameters should be optimized in order to achieve optimum layer-to-layer adhesion (increased contact area and reduction of the 3D-printed induced porosity to the minimum) and increase pieces’ density and strength. The printing feeding mechanisms also impact the printability/finishing of the parts; as well as the usage of in situ controllable drying mechanisms [15].

Revelo et al. [6] used kaolinite clay for AM, applying the DW printing technology. They printed cylinders for compression tests that were analyzed after being cured for one day at room temperature and then heat-treated at 1100 °C for 1 h. This investigation showed that the AM of kaolinite clay-based ceramics is a versatile and simple technology, compatible with multiple applications. This is feasible, even when clay does not undergo any beneficiation process, as is the case with the typical ceramic pastes used in industry. Nonetheless, the finishing quality and mechanical performance of the obtained product were lower in comparison to the cases when optimized and beneficial clay pastes are used. Therefore, when using additives, the finish can be tailored to almost all desired properties for any application, creating new possibilities, not only at an industrial scale but also for small projects. 

A summary of the recent developments in the manufacturing of single and multi-ceramic structures by robocasting was presented by Peng et al. [10]. The authors stated that robocasting is the proper technique to construct fine and dense ceramic structures with geometrically complex morphology.

It should be emphasized that, even being out of the scope of the present work, to highlight controversial and diverging hypotheses or even a simpler comparative analysis of the state of the art, it is difficult to carry out due to the apparent literature scarcity on the AM processes of clay-based materials similar to those under study, which are even more problematic due to the different printing technologies used, different materials nature and workability. Eliza et al. [2] presents a review which shows a growing trend in the publications reporting the study of 3D printing of ceramics, yet regarding clay-based materials such as stoneware, research is still scarce.

### 1.2. Microwave Firing Overview

After forming, to achieve the mechanical strength and aesthetic properties, the green parts are fired using gas or electric resistive heating elements. This firing process typically requires high temperatures and long processing times, which in the case of the present fired products (stoneware), it takes 10 h to reach temperatures around 1200 °C. The 3D printing (slurry-based) of coffee-cup-shaped stoneware was carried out in the search of a novel firing technique that employs MW radiation as the energy source. The MW furnace consists of a closed metallic chamber built with six magnetrons (energy sources). The temperature is controlled by a pre-calibrated pyrometer [16]. Since MW radiation has the potential to penetrate the material being processed, and once the overall firing process is optimized, heat is generated throughout the whole volume of the material. This is impossible to accomplish if considering conventional heating technologies/methods. Due to the volumetric heating, which the MW heating process allows, it is possible to attain faster firing processes without thermal related defects in the parts, such as deformations and cracks formation [17]. One advantage of this technology is the fact that the material becomes the heat source itself.

The sintering process using MW radiation is based on the conversion of the electromagnetic energy into the thermal form. MW heating is caused by the ability of the material to absorb the electromagnetic energy and convert it to heat. The heating potential from MW energy is mainly due to the dipolar polarization mechanism, where the dipole, sensitive to the external electric field, will try to align with it. Under high-frequency electric fields, the dipoles do not have enough time to respond to the sinusoidal field change, resulting in molecular internal friction. That results in energy dissipation with heat generation in the material [18,19,20].

The dielectric properties (namely the permittivity) of the materials are one of the main factors to evaluate the viability of MW heating [18]. The microwaves–matter interaction is governed by the material complex permittivity equation, ε*, given by [18,21]

(1) $$\varepsilon^{*}\left(\omega ,T\right)=\varepsilon^{\prime }\left(\omega ,T\right)-i\varepsilon^{\prime \prime }(\omega ,T)$$

where, *ε′*, the real part of permittivity, also known as dielectric constant, measures the ability of the material to store energy; *ε″*, the imaginary part of permittivity, also identified as dielectric losses, represents the ability of the material to convert the stored energy into heat; ω is the angular frequency of the applied field; and *T* is the material’s temperature.

The dielectric interactions of materials are further described by two parameters, on which the uniformity of heating profile depends: the power generation per volume unit, *P*, and the depth of penetration of microwaves into the material, *D_p_*, are given by [20,21]

(2) $$P=\omega \varepsilon_{0}\varepsilon^{\prime \prime }E^{2}$$

(3) Dp=λ02π2ε′1+ε′′ε′212−1−12

where $\varepsilon_{0}$ is the permittivity of free space ($\varepsilon_{0}$ = 8.854 × 10^−12^ F/m), *E* the electric field strength and $\lambda_{0}$ the wavelength in the free space. If $\varepsilon^{\prime \prime }\leq \varepsilon^{\prime }$, *D_p_* can be approximated (with an error up to 10%) to

(4) $$D_{p}\approx \;\frac{\lambda_{0}\sqrt{\varepsilon^{\prime }}}{2\pi \varepsilon^{\prime \prime }}$$

The material temperature evolution during MW heating is governed by the heat diffusion equation including the volumetric heat generation term [22,23,24].

(5) $$\rho C_{P}\frac{\partial T}{\partial t}=\nabla .\left(k\nabla T\right)+P$$

where *r*, *C_P_* and *k* are the material’s density, specific heat and thermal conductivity (an anisotropic parameter).

Comparing MW with conventional stoneware firing, the main difference, in practical terms, refers to the technology itself, whereby through the interaction with MW radiation, the material can be directly heated. Direct heating, where the material to process is transformed into the heat source, allows a faster process without rejection of parts, presenting advantages over the conventional heating methods, namely the grain growth and densification [25].

Zuo et al. [26] study the conventional and MW sintering of undoped and doped alumina with different amounts of MgO. The thermal routes were identical for both processes, with a heating rate of 25 °C/min until reaching 1550 °C, plus a soaking time of 5 min. MW sintering demonstrates a noticeable enhancement in the densification process and final density compared to conventional sintering of Al_2_O_3_ samples. The research presented by Ramesh et al. [27] compared the mechanical properties and microstructural evolution of yttria-stabilized zirconia sintered by MW and conventional methods. The MW sintering was found to be beneficial in lowering the densification temperature and resulted in enhanced bulk density and improved mechanical properties compared with conventionally sintered samples. Zeng et al. [25] presented a comparative study between MW and conventional sintering of Li_2_TiO_3_–Li_4_SiO_4_ biphasic ceramic pebbles. In both cases, they were fired in a temperature range between 750 °C and 900 °C, for 10 min. The authors stated that the desired phase transformation occurs at a lower sintering/firing temperature by MW heating, the microstructure of the sample exhibits more uniformly distributed grain sizes, and the relative density and crush load of ceramic pebbles are significantly enhanced. Sharma et al. [28], investigated the effect of MW and conventional sintering on the doping behavior of Mn^2+^ and Nb^5+^ in lanthanum germanate based apatite. Microwave-fired apatites at 1400 °C for 30 min presented a higher density and more uniform grain growth than the samples conventionally fired at 1400 °C for 4 h. Moreover, MW-fired apatites showed higher Vickers hardness value, higher conductivity and lower activation energy values than the conventionally fired ones. Lyra et al. [29] studied the sintering of clay ceramics in conventional and MW furnaces. The conventional sintering was performed with a heating rate of 10 °C/min, for 60 min, while the MW sintering was carried out with a heating rate of 50 °C/min, for 5, 10 and 15 min. According to the reported results, the fast-firing, between 5 and 15 min, did not result in the specimen’s damage due to thermal stress. Besides that, for temperatures of 1000 °C and below, the ceramic properties were similar for both firing processes. However, for sintering temperatures above 1000 °C, the MW-fired specimens showed a reduction in water absorption and an increase in the compressive strength and microhardness values. These specimens also presented a microstructure with a reduced concentration of structural defects, indicating greater crystallographic organization, when compared to specimens sintered in the conventional furnace.

Santos et al. [30] reported the lowering of the optimal firing temperature, in the order of 50 °C to 100 °C, in utilitarian microwave-fired stoneware when compared to the conventionally fired samples, with similar and even improved mechanical properties, such as lower water absorption. Microwave firing shows that some samples’ phase transformations occur at lower firing temperatures [31], influencing its color evolution during firing [30].

The present study aims to demonstrate that MW firing of 3D-printed stoneware is a viable option, where it is possible to produce stoneware products with comparable properties as to those obtained when conventionally fired, with the benefits of not inducing defects or cracks and with a reduction of the firing time. Microwave firing is thus presented as an alternative or auxiliary technology for 3D printing stoneware manufacturing, having a strong potential for processing costs reduction and product features improvement. By these ways, two cleaner and environmentally friendly solutions for materials processing were conjugated, namely MW firing and AM technologies.

## 2. Materials and Methods

### 2.1. Stoneware 3D Printing

**Figure 1:**
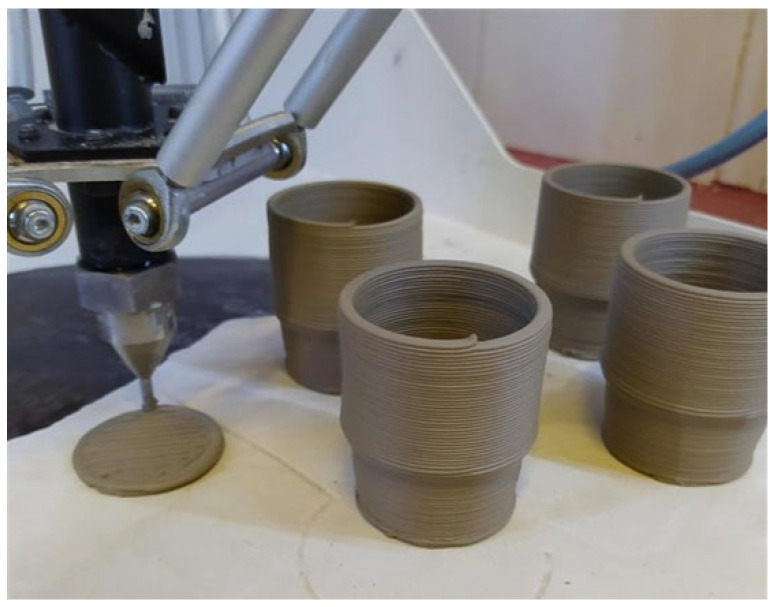
Printing of 3D stoneware pieces with a coffee-cup size and shape.

Stoneware pieces with coffee-cup size and shape were printed using the Delta WASP 40,100 Clay equipment [32]. Figure 1 shows the samples being printed. Material properties and processed parameters are described in Table 1. The material for 3D printing followed the standard means of preparation, more particularly adding the adequate water proportion to the paste until reaching the desired viscosity, a uniform consistency and adequate plasticity to flow through the nozzle at modest–high shear rates without losing its shape retention and creating defects during its multi-layer construction (printing). This allows the material to be easily extrudable, preventing defects such as cracks due to lack of moisture, or layers and full part collapses due to excess of water. The 3D-printed greenware pieces have a water percentage of around 25%. The pieces were created using a Computer-Aided Design (CAD) software (more precisely the Solidworks^®^ 2019), following a G-code programming using the Slic3r slicer software, version 1.3.0. The stoneware paste was acquired from Mota Pastas Ceramicas S.A., from Mota Ceramic Solutions group (Pombal, Portugal) [33].

For mechanical testing, according to the ASTM C1161-13 standard for 3-point flexion, stoneware test bodies with parallelepiped shapes were printed. After firing they have approximately 8 w × 6 t × 90 L mm, where w, t and L represent the test bodies width, thickness and length, respectively.

The microstructure/porosity (µCT) analysis was performed on smaller size samples or pieces having 34 mm in height and 26 mm in diameter (mentioned as smaller samples). They have the same shape and wall thickness, of ~4 mm, as the main pieces presented in Figure 1. The image pixel size in both big (main pieces) and small-size pieces were ~50 µm and 25 µm, respectively. Aliquots with a surface area of 10 mm^2^ and 20 mm^2^ were later harvested from the main samples to attain a better image pixel size. The pixel sizes were 8 µm and of 16 µm for the (10 × 10) mm and (20 × 20) mm size aliquots, respectively. The 20 × 20 mm size aliquots are effectively more representative of the piece’s microstructure.

### 2.2. Microwave and Electric Firing

**Figure 2:**
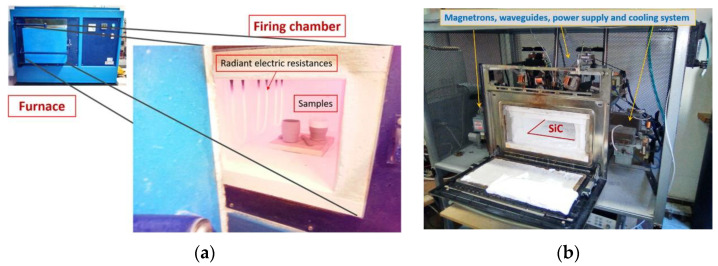
Electric furnace (**a**) and Microwave (MW) (**b**) furnaces.

The 3D-printed stoneware was fired in electric and MW furnaces as shown in Figure 2. The electric furnace is a conventional resistive Termolab S.A. (Águeda, Portugal) chamber furnace MLR type and has a nominal power of 8 kW. The MW furnace is a multi-mode MW furnace, allowing the firing of multi-objects per batch [34,35]. The home-built MW furnace comprises six magnetrons, each with 900 W nominal power, and was built in the Physics Department of the University of Aveiro (Aveiro, Portugal).

Due to the stoneware’s low MW absorption capability, at the base of the MW furnace, a silicon carbide (SiC) plate was used as MW susceptor, helping in the firing of the stoneware [34,36,37]. The SiC plate also serves as the base for the stoneware pieces. The use of MW susceptors is especially relevant when the materials to be heated are poor MW absorbers, such as stoneware (see Table 2), and require some initial temperature boost to reach the material critical temperature (*T_C_*), above which the material significantly starts absorbing the MW radiation. At these initial low temperatures, it is mainly the SiC that absorbs MW radiation and not so much the material to be processed, which receives heat from the MW-heated SiC [38,39].

Also, due to the required high energy and electric field homogenization, allowing the fast firing of the stoneware (without piece deformation and crack formation), a temperature–time dependent permutation (On–Off) control code was developed for magnetrons (MW generators) actuation [25]. For temperature monitoring and control, a pyrometer (calibrated for the stoneware under study) is used. The pyrometer measures the temperature of the central sample closer to the furnace door. A type-S thermocouple installed in the back of the furnace works as a backup/policeman temperature unit sensor. The control of the furnace is made by a home-built Labview code. Along with these temperature sensors, Process Temperature Control Rings (PTCRs) [40] were used to measure the temperature of each piece.

Further information concerning the furnace, power/rate control, temperature measurement and experimental procedure can be found in the literature ([34,35] and in the supplementary file in [31]).

**Figure 3:**
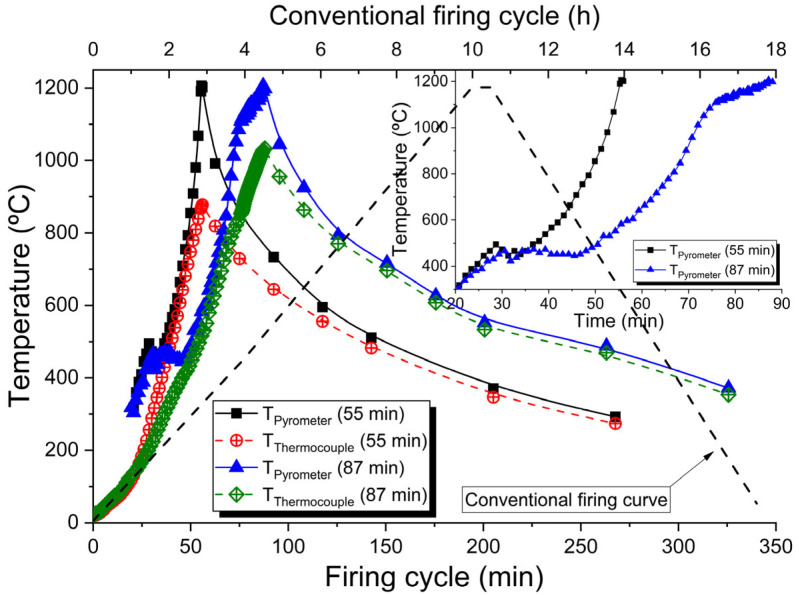
Heating cycles of 55 min, 87 min and 10 h implemented in both MW and electric firings.

The 3D-printed stoneware was fired in both electric and MW furnaces, using 3 different firing curves, up to a maximum temperature of 1200 °C. The 10 h firing curve, only implemented in the electric furnace, followed the protocol conventionally employed in the firing of such products. The respective conventional firing curve is shown (dashed line) in Figure 3, where the secondary *x*-axis is related only to this curve. The pieces conventionally fired are seen as references. In Figure 3, the firing cycles implemented in both MW and electric furnaces are presented. More precisely, the firing cycles obtained in the MW furnace, which were later replicated in the electric furnace for the fast-firing cycles. The fast-firing cycles implemented in the electric furnace are those measured by the pyrometer. Figure 3 inset shows the 55 min and 87 min firing curves with focus only on the heating cycle. It is noteworthy that the use of thermocouples inside MW furnaces causes problems and inaccuracies in temperature measurement [41], which is the reason why the entire process is controlled by the pyrometer, which is measuring the temperature in one of the pieces inside the furnace. As seen in Figure 3, the temperature measured using the pyrometer is above that measured using the thermocouple, mainly above 800 °C. Both curves tend to converge during cooling.

Independently of the heating rate, at approximately 500 °C a change in the heating curve is observed. That is attributed to phase changes of the mineral kaolinite, mainly to its dehydroxylation, which according to [42], starts at 420 °C. This transformation may proceed up to 950–1000 °C as the kaolinite–metakaolinite transformations depend on the thermal treatment [43,44,45]. Those transformations are not detected by the thermocouple as it is measuring the air temperature. The pyrometer is measuring the sample’s surface temperature, and transformations with a temperature response are detected by it.

### 2.3. Methods

After firing, the samples were subjected to visual analysis, followed by flexural strength and porosity analysis.

For mechanical testing, 13 to 15 stoneware test bodies were fired in each furnace, as the main samples, and later subjected to compressive transverse loading. More specifically, flexural assessment using 3-point bending test. The Instron^®^ model 4505 universal testing machine was used with lower spans of 9 mm and a crosshead speed of 1 mm/min. Tests were performed based on the test conditions for type C specimens as described in ASTM C1161-13 standard for advanced ceramics [46].

The 3D morphology and porosity analyses were performed using X-ray microcomputed tomography (µCT) equipment from SkyScan 1275 (Bruker µCT, Kontich, Belgium) with penetrative X-rays of 80 kV and 125 µA, in high resolution mode, 42 ms of exposure time, 3 of frame averaging, 0.50 deg of rotation step, 1 mm Al filter and 360° of rotation. NRecon (v.1.7.3.1 software, Bruker, Kontich, Belgium) and CTVox (v.3.3.0 r1403 software, Bruker, Kontich, Belgium) software were used for 3D-reconstruction and CTAn software (v.1.17.7.2 software, Bruker, Kontich, Belgium) was used in morphometric analysis (e.g., porosity values and pore size distribution), in which the images were segmented and analyzed. Herein, the thresholding or image binarization/segmentation was performed using a global thresholding technique for each sample, which employs a fixed range of greyscales (e.g., lower and upper scales set at 78 and 255 for “Ref.” sample) for both the foreground (white) and the pixels outside of the range area, which are set as the background (black).

Samples complex permittivity measurements were performed at stoneware paste (greenware), dried greenware and greenware electrically fired at 1200 °C for 10 h (stoneware reference—Ref.). The samples complex permittivity measurements were performed at room temperature using the resonant cavity method, operating at 2.7 GHz, and applying the small perturbation theory [47]. In this method, the resonance peak frequency and the quality factor of the cavity, with and without a sample, are used to obtain the complex permittivity of the material. The shift in the resonant frequency of the cavity, *Δf* = *f_0_*− *f_S_*, caused by the insertion of the sample, is related to the real part of the complex permittivity, $\varepsilon^{\prime }$, and the change in the inverse of the quality factor of the cavity, *Δ(1/Q)* = *1/Q_S_* − *1/Q_0_*, gives the imaginary part, $\varepsilon^{\prime \prime }$. Considering only the first-order perturbation in the electric field caused by the sample, and separating the real and imaginary parts of the complex permittivity, $\varepsilon^{\prime }$ and $\varepsilon^{\prime \prime }$ can be obtained by

(6) $$\varepsilon^{\prime }=K\frac{f_{0}-f_{s}}{f_{0}}\frac{V}{v}+1$$

(7) $$\varepsilon^{\prime \prime }=\frac{K}{2}\left(\frac{1}{Q_{S}}-\frac{1}{Q_{0}}\right)\frac{V}{v}$$

where *K* is a constant related to the depolarization factor, which depends upon the geometric parameters, and where the indexes *0* and *S* refer to the empty and loaded cavity, respectively. *V* and *v* are the cavity and sample volumes, respectively. Using a material of known complex permittivity, the value of *K* can be calculated. In this study, polytetrafluorethylene (PTFE) was used with the same size and shape of the samples. More information regarding complex permittivity measurements can be found in [48,49,50,51].

For a better perception of the vicissitudes and complexity of microwave processing of materials, as experimentally we cannot look inside the furnace, simulations were carried out using the COMSOL Multiphysics software, version 5.2a. As experimentally performed, the magnetron frequency of 2.45 GHz and power of 900 W were considered in the simulations.

## 3. Results

Before the presentation and analysis of the experimental results, a comparison of the MW fast-fired samples with electrically fast-fired and electrically conventionally fired samples is presented in the samples’ permittivity assessment (Section 3.1), from where it is realized that both dried greenware and stoneware presents very low MW radiation absorption capability at room temperature. These results agree with the ones presented in [39], where a comparable material to stoneware was studied, presenting very low $\varepsilon^{\prime }$ and $\varepsilon^{\prime \prime }$ at room temperature, increasing above the sample’s critical temperature, *T_C_*, of ~700 °C. Figure 3 shows that the temperature measured using the pyrometer is above the one measured using the thermocouple, mainly above 800 °C. That might be due to the high MW absorption capability owed to the change of the permittivity of the stoneware with the increasing of its temperature. From the behavior of both temperature curves, the stoneware’s critical temperature is estimated at around 800 °C.

In Section 3.2, some simulation case studies are briefly presented, highlighting aspects that must be considered when using MW technology for material sintering. This elucidates some of the experimental results and demonstrates the need for an accurately controlled process to achieve a more volumetric heating. In Section 3.3, the comparative study of MW versus electric stoneware firing is presented.

### 3.1. Samples Complex Permittivity

Table 2 shows the complex permittivity values for stoneware and SiC. It is worth mentioning the relatively high permittivity for both greenware (containing ~25% water in its composition) and SiC, and the low permittivity of the dried greenware and stoneware samples.

**Table 2:** Samples complex permittivity measured at ~2.7 GHz at room temperature.

Sample	Dielectric Constant (*ε′*)	Dielectric Loss (*ε″*)
Greenware	10.43	0.94
Dried greenware	3.67	0.30
Stoneware	4.21	0.07
SiC	10.74	0.74

### 3.2. Microwave Firing Simulation

**Figure 4:**
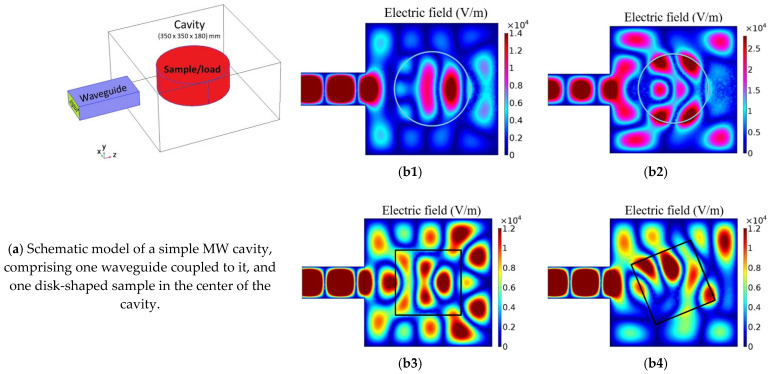
(**a**) Schematic model of a MW cavity and simulation of samples’ effects on the electric field (V/m) pattern for (**b1**) a disk sample with volume *V_0_*, (**b2**) a disk sample with volume = 0.9 *V_0_*, (**b3**) a parallelepiped sample with volume *V_0_*, and (**b4**) the parallelepiped sample in (**b3**) rotated 12.5°.

First, by presenting a simple model, Figure 4, it is shown that MW firing is a highly complex technology, as the heat wave or the heat created in the piece is dependent not only on the MW energy, on the sample’s dielectric properties and radiation frequency, but also on the pieces size, form, and relative position of them inside the MW furnace.

**Figure 5:**
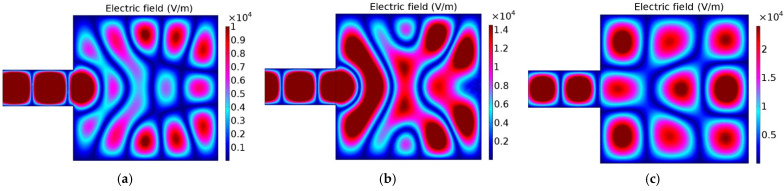
Electric field (V/m) pattern as a function of the MW cavity size. Only empty cavities considered. (**a**) Cavity size: 350 × 350 × 120 mm; (**b**) Cavity size: 350 × 350 × 150 mm; (**c**) Cavity size: 350 × 350 × 180 mm.

In Figure 4, the effect on the electric field pattern (maxima and minima of energy) is clearly visible due to the volume change of the sample, for example, due to the shrinkage during firing. The electromagnetic field pattern also changes due to the shape and position of the sample inside the MW furnace, as shown in Figure 4, and due to changes of the cavity size, depicted in Figure 5. Not only does the electromagnetic field pattern change, but also the field intensity changes, increasing ~two times (visible in both Figure 4b1 and 4b2 and in Figure 5a–c). Figure 5c is also comparable to all cases presented in Figure 4, as the volume of the cavity is the same, only differing in being devoid of a sample. It should be noted that different color scales were used because it is intended to highlight the existing pattern/difference between this numerical case studies, and because if the same scale of Figure 4b2 is adopted in Figure 4b1, Figure 4b1 becomes difficult to analyze since it becomes highly bluish, losing definition.

**Figure 6:**
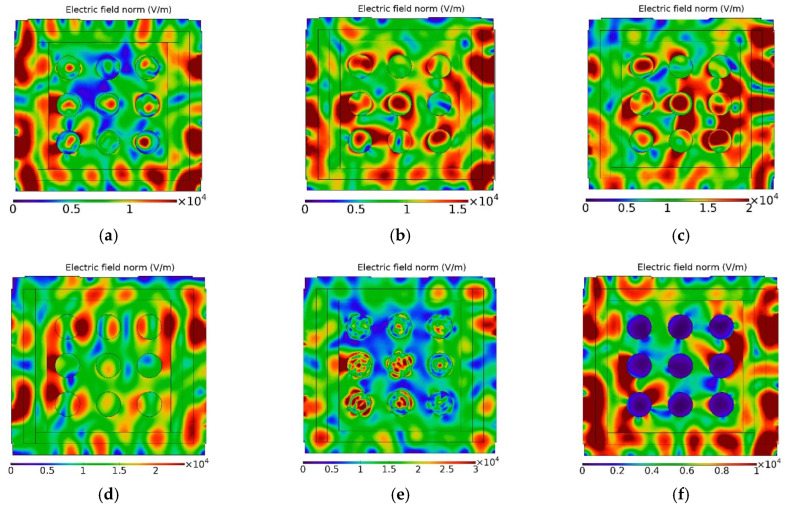
Simulation of the electric field (V/m) pattern for several $\varepsilon^{\prime }$ and $\varepsilon^{\prime \prime }$ conditions; (**a**) Greenware; (**b**) Dried; (**c**) Stoneware; (**d**) $\varepsilon^{\prime }=2.0$ and $\varepsilon^{\prime \prime }=0.01$; (**e**) $\varepsilon^{\prime }=30.0$ and $\varepsilon^{\prime \prime }=0.01$; (**f**) $\varepsilon^{\prime }=2.0$ and $\varepsilon^{\prime \prime }=15.0$.

In Figure 6, another simulation case study is shown, where the effect of the change of the samples dielectric properties is presented, from the greenware state to the stoneware product. This case study considers nine cylindrical samples inside the microwave furnace, with the same size and shape, and distributed in the same positions as in the real microwave furnace/experimentation performed with the 3D-printed stoneware samples, whose results are presented in Section 3.3.

Comparing Figure 6b,c, it is seen that relatively small changes in both $\varepsilon^{\prime }$ and $\varepsilon^{\prime \prime }$ can result in changes in the electromagnetic field pattern.

Other (hypothetic) case studies are presented, Figure 6d–f. Comparatively to Figure 6d, a high number of maximums of energy are obtained in samples presenting a low dielectric loss ($\varepsilon^{\prime \prime }=0.01$) and a high dielectric constant ($\varepsilon^{\prime }=30.0$) (Figure 6e). A low penetration depth is observed in samples with high dielectric losses ($\varepsilon^{\prime \prime }=15.0$) (Figure 6f).

**Figure 7:**
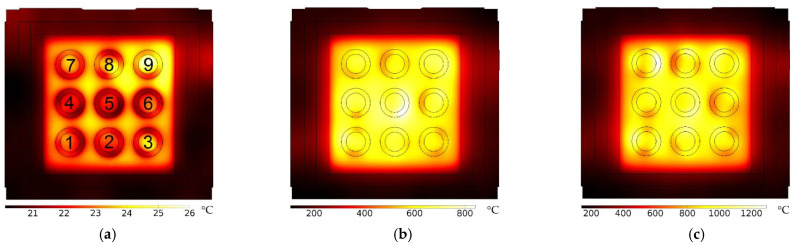
Spatial temperature simulation inside the MW furnace, having 9 samples distributed as in the experimental part. (**a**): 15 min of firing. (**b**): 40 min of firing. (**c**): 60 min of firing.

Along with the simulation of the electromagnetic field pattern, the temperature evolution inside the furnace with 9 samples was evaluated. Simulation results are presented in Figure 7. The simulation protocol followed an equivalent On–Off permutation of the magnetrons, as experimentally performed.

**Figure 8:**
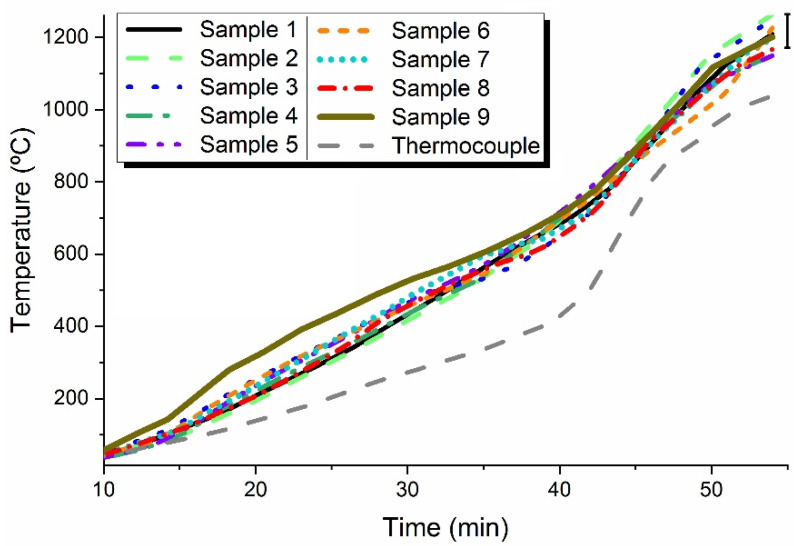
Simulation of the temperature in each MW-fired sample. The right vertical black bar (at ~55 min) embodies the minima and maxima of PTCR’s temperatures (see below in Table 3 the corresponding temperatures). The first 10 min are not shown since no significant information is provided.

In this simulation, the “pyrometer” is measuring the temperature of the central sample closer to the furnace door, as experimentally performed (sample number 2 in Figure 7a). Figure 8 shows the simulation of temperature evolution for each of the 9 samples and where the thermocouple tip should be.

It is noteworthy that present simulations are only demonstrative of a hypothetic and illustrative situation related to MW firing, as the permittivity dependency with the temperature (as the pieces are heated) is unknown. Both the dielectric constant and the loss data, used as inputs in the COMSOL software, version 5.2a, were engineered in order to obtain a heating process equivalent to that obtained experimentally (Figure 3), which is to attain a firing temperature of around 1200 °C in ~1 h.

### 3.3. Experimental (Microwave and Electrically Fired Samples)

After firing, and as usual, the mechanical and aesthetic properties were evaluated, with rupture energy evaluated in test bodies, or specimens, and not in the stoneware coffee cups (main samples).

#### 3.3.1. Aesthetic Evaluation

**Figure 9:**
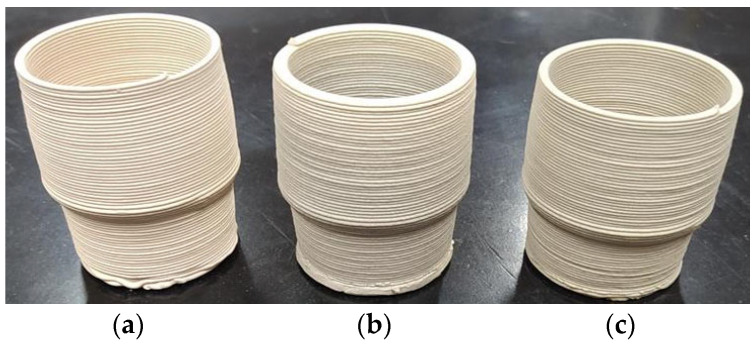
Color of microwave and electrically fired samples; (**a**) Electrically fired for 70 min; (**b**) MW-fired for 55 min; (**c**) Electrically fired for 10 h.

Stoneware pieces with the shape of a coffee cup were evaluated essentially in terms of their aesthetic properties. As presented in Figure 9, samples fired in the MW furnace at 1200 °C for 55 min present a color (when not decorated) closer to the reference samples (electrically fired at 1200 °C for 10 h) than to the samples electrically fired at 1200 °C for 60 °C. Both microwave and electrically fast-fired (for <60 min) samples present a lighter color than the reference. Electrically fast-fired samples are creamier in color, whereas electrically conventionally fired are grayer. The MW-fired samples lay between the two electrically (fast-fired and conventionally fired) samples’ colors.

It should be mentioned that no de-bonding was observed after printing, neither during microwave or electrical firing, indicating excellent adhesion between layers. Samples that were electrically fast-fired presented some deformation and crack formation at the sample’s base. 

Aesthetic properties were also evaluated in partially and fully glazed samples fired for ~90 min using both furnaces.

**Figure 10:**
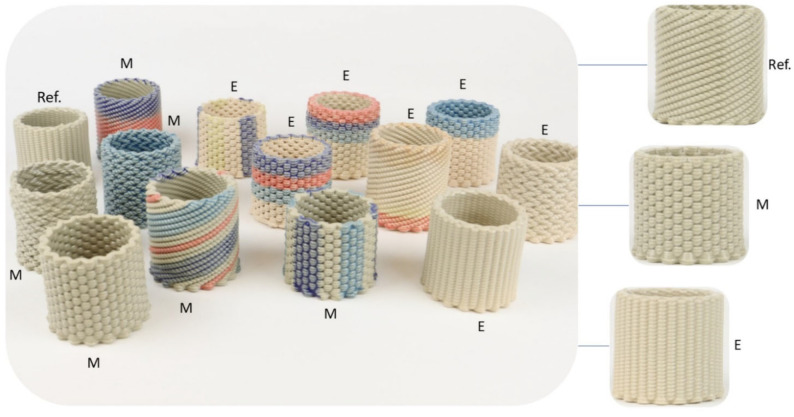
Photograph of samples that were microwave fast-fired (M) in 87 min, electric fast-fired (E) in 87 min and electric conventionally fired (Ref.) in 10 h at 1200 °C. In the individualized images (on the right), the grayer color of M and Ref. samples and the creamier color of E sample are highlighted.

**Figure 11:**
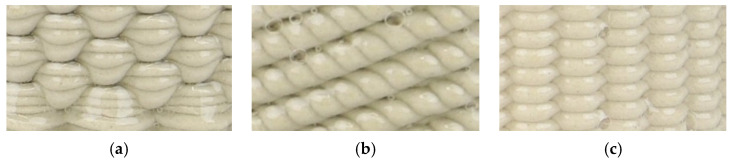
Photograph of (**a**) (M) microwave fast-fired (87 min), (**b**) (Ref.) electric conventionally fired (10 h) and (**c**) (E) electric fast-fired (87 min), fully glazed samples at 1200 °C: highlighting of the trapped bubbles in the transparent glaze.

A representative set of microwave and electrically fired samples are shown in Figure 10 and Figure 11. The MW-fired samples do not present any visible defects or significant differences when compared with the reference, whereas electrically fast-fired samples present a lighter/creamier color, as before. In Figure 11, the trapped bubbles in the transparent glaze of microwave and electrically fired samples are highlighted.

#### 3.3.2. Spatial Temperature Evaluation

As the temperature is not the same for all the samples inside the furnace, as shown in Figure 7 and Figure 8, the temperature measurement has been the subject of study using Process Temperature Control Rings (PTCRs) of the LTH − 152 series, covering the temperature range from 970 °C to 1250 °C [40]. To reach a reliable and representative temperature of the pieces, one PTCR element was introduced inside each test piece.

By quoting [40], ‘A PTCR is a ceramic device that registers the total amount of heat transferred to it. Because of the advanced technique and materials applied and used for this product, it gives a fair representation of the real heating process taking place in a kiln at the location of the ring’.

Using the measurement of the retracted ring, after firing, a temperature value (ring temperature) can be attributed. For example, the PTCR fired using the conventional 10 h heating curve (sample Ref. in Figure 10), presented a diameter of 19.29 mm, that according to the PTCR-ETH manufacturer conversion table, corresponds to a ring temperature of 1203 °C.

Table 3 presents the PTCR diameter and the PCTR temperatures used during electric and microwave firings. Data were determined according to the manufacturer’s conversion table, which considers firing curves normally implemented in the industry. Therefore, the real PCTR temperatures need to be recalibrated assuming the faster firing cycles implemented in the present study. As equivalent heating curves were used in the MW and electric test firings, the PTCR fired in the electric furnace at 1200 °C for ~60 min, which presents a diameter of 19.84 mm, must correspond to a temperature of 1200 °C, and not 1097 °C. The ring temperature of 1097 °C was determined considering the manufacturing conversion table. The corrected PTCR temperature is also reported in Table 3.

Firing tests present a very good agreement between the temperatures measured using PTCR elements and the pyrometer, with maximum differences of the order of 70 °C between samples, and an average PTCR temperature, and respective error, of (1207 ± 15) °C. The PTCR element positioned in the piece where the pyrometer is measuring, and controlling the firing process, measures 1215 °C when the pyrometer measures 1200 °C, a residual difference of 15 °C (corresponding to an error of only 1.25%).

**Table 3:** Firing methods, ring temperature and ring-corrected temperature.

Firing Method		Ring Diameter (mm)	Ring Temperature (°C) ^1^	Ring-Corrected Temperature (°C)
Electric	10 h (Ref.)	19.29	1203	-
	60 min	19.84	1097	1200
	87 min	19.80	1109	
Microwave ^2^	1	19.84	1097	1200
	2	19.79	1112	1215
	3	19.87	1086	1189
	4	19.62	1153	1256
	5	19.86	1090	1193
	6	19.78	1115	1218
	7	19.83	1100	1203
	8	19.82	1103	1206
	9	19.89	1079	1182

^1^ Determined accordingly to the PTCR-ETH manufacturer conversion table. ^2^ Fired for 60 min. The numbers 1 to 9 are the position of the samples inside the MW furnace, as represented in Figure 7a.

#### 3.3.3. Flexural Tests

**Figure 12:**
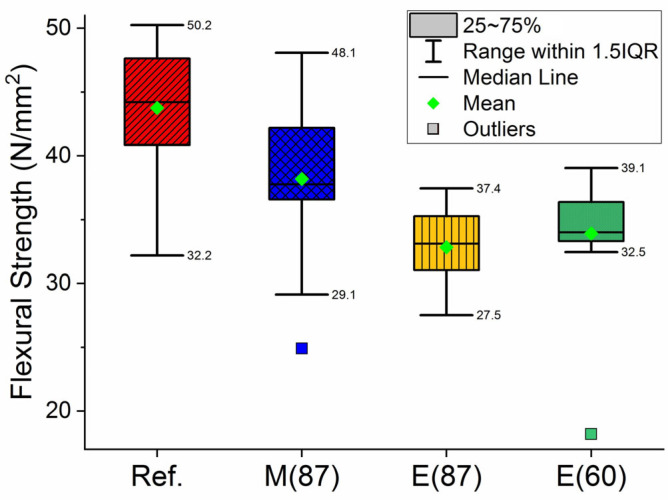
Boxplots of reference samples, Ref.; microwave fired, M(87), and electrically fired samples for 87 min, E(87); and electrically fired samples for 60 min, E(60). Firing temperature of 1200 °C.

Flexural tests of the best 10 samples are presented in Table 4. Figure 12 shows the flexural strength boxplots of the full sampling, of 13 to 15 samples, for each fired batch. From both Table 4 and Figure 12, it is observed that MW-fired specimens required a higher load to break when compared with electrically fast-fired samples, both having lower strength compared to the reference samples. From Figure 12, it is also observed that MW-fired samples’ flexural strength have an overall greater proximity to the reference than the electrically fired ones.

#### 3.3.4. Morphology and Porosity/µCT Evaluation

**Figure 13:**
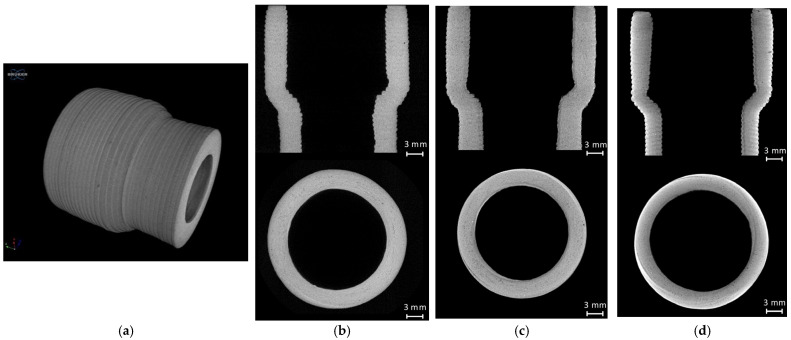
µCT images of the reference, MW and electrically fired samples with 34 mm in height and 26 mm in diameter; (**a**) A 3D µCT reconstructed image; (**b**) Electrically fired sample with a total porosity of 1.4%; (**c**) Microwave fired sample with a total porosity of 1.5%; (**d**) Reference sample, with a total porosity of 1.9%.

As mentioned in Section 2.3, the porosity of smaller samples (34 mm height and 26 mm diameter) was also analyzed. Figure 13 presents the representative 3D and 2D µCT images of the reference (1200 °C in 10 h), MW and electrically fired samples at 1200 °C for 87 min, showing that in all cases, more than 98% of the pores are smaller than the image pixel size resolution of 25 µm.

**Figure 14:**
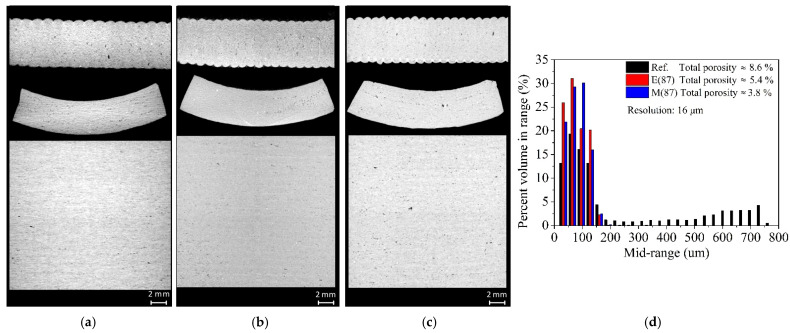
µCT analysis of the reference, MW and electrically fired aliquots with 20 mm^2^ and a wall thickness of ~4 mm; (**a**) Electrically fired aliquot with atotal porosity of 5.4%; (**b**) Microwave fired aliquot with a total porosity of 3.8%; (**c**) Reference aliquot with atotal porosity of 8.6%; (**d**) Aliquots percent volume porosity size.

Figure 14 presents the representative 2D µCT images of the 20 mm^2^ aliquots harvested from the reference, MW and electrically fired main samples at 1200 °C for 87 min, providing information about the pore size distribution profile (i.e., mid-range vs. percent volume) and porosity values. The images with a pixel size resolution of 16 µm were attained for the aliquots, as previously mentioned in Section 2.3. Total porosities of 3.8%, 5.4% and 8.6% were determined for MW-fired, electrically fast-fired (87 min) and conventionally fired (10 h) samples. From the histogram (Figure 14d), the bigger pore sizes in the case of conventional fired samples are clearly visible, being almost absent in the fast-firing cases.

## 4. Discussion

Briefly, simulation results show that the MW firing process is a highly complex process, with accurate temperature measurements and control being one of the critical issues. As shown in Figure 8 and in Table 3, different temperatures are observed between MW-fired samples. That is also observed in electrically and conventionally (gas) fired products. These results also show that the thermocouple, which is measuring the temperature in air, is measuring a temperature below that of the samples, as expected since the samples are transformed in the heat source by themselves due to the interaction with MW radiation. The faster the heating, the higher the difference between the real temperature (pyrometer) and the one measured by the thermocouple. From Figure 3, when the samples are fired for 55 min, and when the pyrometer measures 1200 °C, the thermocouple measures around 880 °C. When fired for 87 min, and the pyrometer measures 1200 °C, the thermocouple measures around 1030 °C. The use of a thermocouple might also influence the electromagnetic field and create a field enhancement effect around it [52]. The simulation results, Figure 8, also show that at the end of the heating cycle, the differences in temperature between samples are of the order of those presented in Table 3. The vertical black bar in Figure 8 embodies the minima and maxima of the PTCR’s temperatures of the order of 70 °C, and the simulation results of the order of 115 °C. This comparison must be performed carefully as the simulation is only representative of a hypothetical set of input conditions. The simulation does not consider the materials’ phase transformations, the thermal conductivity, nor volumetric changes due to shrinkage. The $\varepsilon^{\prime }(T)$ and $\varepsilon^{\prime \prime }(T)$ input curves are mainly considered hypothetical. For a correct prediction and superior control of the material processing by MW firing, it is not enough to know the materials properties and how they change with temperature. The presence or absence of moisture, the raw materials composition, the phase transformations that occurs during firing and the material’s thermal properties (as a function of the temperature) are also of great relevance, mainly those intrinsic to the thermal process and important to minimize effects such as thermal runaway [53].

It is noteworthy that during the firing of stoneware, several physical and chemical transformations occur, influencing not only its mechanical and physical properties, but also its color. Compared to the reference and MW-fired samples, electrically fast-fired samples present a lighter color. That is attributed to the slower phase transformations occurring during electric/conventional firing methods [30]. As the stoneware densifies, a more vitreous phase is formed, and during this process, stoneware goes from a grayish color (greenware samples) to creamier (more or less densified) and back to a grayish color again when fully densified (as observed in reference sample in Figure 9c and in [30]). A detailed explanation and comparison between MW and electrically fired stoneware phase transformations can be found elsewhere [31]. 

Experimental data show that the properties of MW-fired stoneware, such as shrinkage (empirically evaluated) and mechanical strength, are comparable to conventionally fired samples, closer to these ones than to the electrically fast-fired samples. Considering the standard deviation (std.) and even the data range distribution within the 1.5 inter-quartile-range (IQR) (Table 4 and Figure 12), mechanical strength for all case studies is in the range or close to the limits.

Microwave fast-firing products reached equivalent characteristics without compromising its quality and features, mainly at the aesthetic level, whereas electrically fast-fired (less than 87 min) samples were not able to fully accomplish the stoneware reference (10 h firing) properties, with a focus on samples’ color. Data not presented here show that temperatures closer to 1300 °C are required for electrically fast-fired samples to reach a color similar to that of MW-fast-fired and conventionally fired samples. Also, electrically fast-fired products present some visible cracks and deformation at the cup’s base. 

Porosity (µCT) results, Figure 13 and Figure 14, reveal that the MW-fired samples have lower porosity and consequently, they are expected to present higher density than the electrically fast-fired and reference samples. Taking into consideration that the flexural strength of stoneware reaches its maximum when the minimum porosity is reached [54], present results seems to be inconsistent with that premise. Porosity values (reference 8.6%, electric-fast-fired 5.4% and MW 3.8%, Figure 14) are not directly correlated with the flexural strength values of 45.7 ± 3.6 N/mm^2^, 34.0 ± 2.1 N/mm^2^ and 40.9 ± 4.4 N/mm^2^ for reference, electric-fast-fired and MW-fired samples, respectively (Table 4).

Although during the scan some effects were minimized and partially corrected using an aluminum filter, it is worth noting that the *µ*CT analysis was not proficient for “bigger” size samples, and because of that, aliquots were used to better see and quantify the smaller size pores. Only residual percentages, lower than 2–3%, independently of the used heating technology, were quantified in both the main and smaller geometric size samples (Figure 13). The same percentage values were attained for the 10 mm^2^ size aliquots. For the 20 mm^2^ size aliquots, porosity values of ~9%, 5% and ~4% were quantified in reference, electrically fast-fired and microwave fast-fired samples, respectively. These values are in accordance with the closed porosity, <10%, for the conventionally fired porcelain stoneware tile products [55,56]. For porcelain tile, it was found that closed porosity values ranged from 10% up to 20% [57]. One must consider that during firing, a time–temperature-dependent densification process occurs during which the smaller size pores coalesce into bigger pore sizes, and the somewhat scarce information concerning lower size porosity values (lower than 16 µm) could be a constraint and a limitation when it comes to a fully porosity evaluation. Nevertheless, the *µ*CT data present valuable information. As seen in Figure 14d, a significantly higher number of bigger size pores are found in the reference sample when compared with both the electrically and MW fast-fired samples. This is a result of a higher residence time inside the furnace during firing (~10 h). Regarding the electrically fast-fired samples, all data (with emphasis on the samples color) are indicative of a not fully fired body, even considering its lower total porosity. Based on the available data, and having in mind what others reported, concerning the effect on grain growth and greater crystallographic organization during microwave processing [21,25,28,29] (see Section 1.2), one can infer that faster microwave firing processes could potentiate the attainment of stoneware products with comparable features as those conventionally fired in less time and with lower pore sizes. More homogeneous microwave heating allows for faster firing processes, which potentiate faster crystallochemical transformations of the stoneware raw-materials [31], as well as the densification, not giving time for the pores to coalesce. Differently from what has been reported in conventionally conformed and MW-fired samples, Section 1.2, [29,30,31], and regarding the inconsistencies related to the premise of obtaining a greater flexural strength when the minimum porosity is reached [54], the present results could be attributed to lower (not presently evaluated) adhesion between layers when fast-fired processes are implemented.

Regarding the overall appearance, compared to the reference samples, and differently to what was reported in conventionally conformed and MW firing of utilitarian stoneware [30], trapped bubbles in the glaze are not significant and the samples presented a smooth surface. The trapped bubbles appear to be dependent on the glazed surface area; samples with a greater unglazed surface area presents fewer trapped bubbles (qualitatively evaluated). That was expected since the gases formed during firing do not find a physical barrier formed by the glazed layer upon the stoneware body surface. Peculiarly, and overall, a larger number of trapped bubbles in the glaze were found in conventionally fired samples. Such results were not expected, as longer firings allow for the gases to penetrate through the glaze and be released. In [30], the need for longer firing cycles in conventionally conformed and microwave-fired samples was verified, so that the glaze does not present bubbles. The referred firing cycles are ~3.3 h, as conventionally implemented in those products [30]. In [58], a better glaze quality was reported, having fewer voids and smoother surface, for dental microwave-fired porcelain compared with conventionally fired products. Taking these considerations, the present results raise some questions of the effect of different glazes for MW firing, which is another variable that should be studied to boost the MW firing as a faster and alternative firing method for dinnerware, artistic and exhibition ware-like products.

In the future, a finer and complete microstructure and morphology analysis of samples should be performed to better explain the observed properties and reached features, and the reason behind the observed differences comparatively to conventional methods of firing. Microwave-fired samples at lower temperatures should also be studied to assess the potential of the temperature reduction as presented by a vast microwave community [18,21,37]. 

It is noteworthy that all samples, including the specimens for the flexural strength test, were manufactured using the robocasting technique, which has some restrictions when it comes to the production of ceramic pieces with a high accuracy. Several factors, as mentioned in the Introduction [5,15], can influence the final shape of the pieces, regardless of using the same G-code; factors related to the process itself, such as pressure changes, which influence the deposition flow rate and might create an irregular and uneven printing, influencing the final shape of the product. These issues might result in significant differences between samples, thus influencing the results. Regardless of some technical issues of the 3D printing technique, the main objective was accomplished—the microwave fast-firing of 3D-printed stoneware parts, without compromising the main characteristics of the final product.

As a final remark, although the tensile forces in the pieces have not been evaluated, it is worth mentioning the absence of visual deformations and micro- and macro-cracks in all MW-fast-fired pieces, including those with a more complex and intricate structure. Theoretically, it was anticipated that these complex, intricate and non-homogeneous structures would present non-homogeneously distributed volumetric stresses and tensile forces that could lead to piece deformation when fast-fired, especially for MW-fired samples due to the way microwave radiation interacts with the matter. Nonetheless, when the microwave firing process is fully controlled, more homogeneous heating is achievable, allowing faster firing processes, which are impossible by conventional means.

## 5. Conclusions

When compared with the 10 h conventionally fired samples (reference), MW-fast-fired samples (in 10–15% of the conventional processing time currently in practice) reached similar product characteristics, without compromising its quality and features, even presenting slightly lower strength. Microwave and electrically fired 3D-printed stoneware present flexural strengths that are 10% and 25% lower than the reference samples, respectively. The aesthetics, mainly by the evaluation of color analysis, disclosed some signs that the stoneware phase transformations were not fully achieved when the samples were electrically fired, as they presented a lighter (creamier) color than both MW-fired and reference samples, with these ones presenting a grayer color. The samples that were microwave-fired presented a color similar to the reference, a sign that they attained equivalent densification, and have ½ of the total porosity of the conventionally fired samples, presenting a relatively higher number of smaller size pores, contrasting with the relatively higher number of bigger size pores found in the reference. Microwave fast firing is presented as a promising and alternative heating technology for prototyping and manufacturing of 3D-printed stoneware. The present study demonstrates that it is possible to produce multi-objects without defects such as deformations and cracks (mainly responsible for piece rejection) using microwave firing technology combined with 3D printing of complex parts that would otherwise be very expensive to produce by applying traditional forming processes.

## Figures and Tables

**Table 1:** Material and printing specifications.

Sample	Stoneware—STW90iEX
Humidity (%)	25
Particle size distribution D(50) (µm)	9.8
Residue (45 µm) (%)	3.7
**3D printing specifications**	
Nozzle diameter (mm)	3
Layer height (mm)	2.5
Layer width (mm)	~4
Scanning speed (mm/s)	23

**Table 4:** Compression strength of reference, MW and electrically fired specimens at 1200 °C for 60 min and 87 min.

Firing Method	Firing Data	Flexural Strength (N/mm^2^)	
		Value	Std.
Electric	10 h (reference)	45.7	3.6
	60 min	35.8	2.1
	87 min	34.0	2.1
Microwave	87 min	40.9	4.4

## Data Availability

Not applicable.
